# The Need for Dynamic Clinical Guidelines: A Systematic Review of New Research Published After Release of the 2017 ATA Guidelines on Thyroid Disease During Pregnancy and the Postpartum

**DOI:** 10.3389/fendo.2020.00193

**Published:** 2020-04-07

**Authors:** Allan C. Dong, Mary D. Stephenson, Alex Stewart Stagnaro-Green

**Affiliations:** ^1^Department of Obstetrics and Gynecology, University of Illinois College of Medicine at Chicago, Chicago, IL, United States; ^2^Department of Obstetrics and Gynecology, Advocate Lutheran General Hospital, Park Ridge, IL, United States; ^3^Department of Medicine, Obstetrics & Gynecology and Medical Education, University of Illinois College of Medicine at Rockford, Rockford, IL, United States

**Keywords:** hypothyroidism, autoimmunity, thyroid, pregnancy, guidelines

## Abstract

**Background:** The American Thyroid Association Guidelines on Thyroid Disease During Pregnancy and the Postpartum (ATA Guidelines) were published in 2017, with an update not expected for another 5 years. Since release of the 2017 ATA Guidelines, greater than 500 articles have been published in the field. Furthermore, there are presently 14 prospective, interventional trials in progress registered at Clinicaltrials.gov Static guidelines updated every 5–7 years fail to provide timely evidence-based guidance to practicing clinicians. Consequently, guideline development should move toward the creation of dynamic documents. The present article reviews the literature published since the 2017 ATA Guidelines, both to benefit clinicians in practice and to make the case for Dynamic ATA Guidelines.

**Methods:** Using the search terms “thyroid” and “pregnancy,” a systematic review of literature published in Pubmed from 3/1/2017 to 12/31/2018 was conducted. The titles and/or abstracts of all articles were reviewed. All articles were classified by subject headings used in the 2017 ATA Guidelines. English-text articles classified under “hypothyroidism” or “thyroid autoimmunity” were examined in full-text. Using the questions and recommendations put forth by the previous ATA Guidelines, relevant articles were selected for discussion in this review.

**Results:** At the time of the search, 659 unique articles on “thyroid and pregnancy” were identified, including 66 original studies on hypothyroidism and 26 on thyroid autoimmunity. Of these, 26 studies on hypothyroidism and 18 studies on thyroid autoimmunity were selected for inclusion in this review based on specific questions in the 2017 ATA Guidelines. Based on these 44 articles, we propose two specific changes to the 2017 ATA Guidelines.

**Conclusion:** Based on new research, we recommend the 2017 ATA Guidelines be updated to recommend against treating thyroid antibody-negative women diagnosed with subclinical hypothyroidism in the second trimester or later; to reflect new, moderate-quality evidence supporting the treatment of thyroid peroxidase antibody-negative women with elevated thyroid stimulating hormone levels in the first trimester or earlier; and to recommend against treatment of euthyroid, thyroid peroxidase antibody-positive women undergoing assisted reproductive technology. Transitioning to a Dynamic ATA Guidelines would allow for these and future recommendations to be implemented in real time.

## Introduction

### Rationale

Pregnancy is a complex endocrinologic and immunologic process that has wide-reaching effects on thyroid hormone homeostasis. Increased estrogen levels result in a 50% elevation of thyroxine binding globulin with a concomitant 50% increase in total thyroxine (TT4) and triiodothyronine. Placental production of human chorionic gonadotropin (hCG) drives a decrease in the thyroid stimulating hormone (TSH) upper limit of normal due to cross reactivity of hCG at the TSH receptor. These critical adaptations in pregnancy are necessary for a healthy pregnancy and a healthy baby. However, the normal physiological changes in thyroid hormone levels make it difficult to distinguish between normal and abnormal hormonal values. The ability to accurately diagnose thyroid hormone abnormalities has increased in importance over the last two decades as ongoing research has linked thyroid hormone disturbances to miscarriage, preterm delivery, gestational hypertension, gestational diabetes, preeclampsia and decreased IQ in the offspring (1).

Clinical guidelines published by national and international societies help providers diagnose and treat thyroid disease in pregnancy. In 2011, the American Thyroid Association (ATA) published comprehensive Guidelines on Thyroid Disease During Pregnancy and the Postpartum (ATA Guidelines), covering more than 20 years of published literature from 1990 up to 2011, along with seminal works pre-dating 1990 (2). Upon release of the 2011 ATA Guidelines, the goal was for a revision to occur within 4–5 years of publication. Nevertheless, new guidelines, which included studies up to the guideline publication date, were not published by the ATA until March of 2017 (3). During that interval, the literature in the field increased rapidly. Specifically, the 2017 ATA Guidelines included citations to 621 references, nearly doubling the 319 references cited in 2011 (2, 3).

Thyroid and pregnancy research continues to be published at a rapid pace. A Pubmed search on “thyroid and pregnancy” reveals over 500 articles that were published in 2017 and 2018. An increasing number of interventional trials are ongoing, promising to yield critical data on the impact of levothyroxine (LT4) therapy in pregnant women with subclinical hypothyroidism and euthyroid autoimmune thyroid disease. It is apparent that a 6-year cycle between Guidelines is too lengthy in which to keep providers up to date. Guideline generation needs to change from a static document to a dynamic document which quickly responds to the publication of new, high-quality research. In a 2014 systematic review of methodological handbooks for clinical practice guideline development, Vernooij et al. concluded that the optimal timeframe between publication of a guideline and the start of an updating process was 2–3 years (4). Based on this conclusion, the updating process for the next version of the ATA Guidelines should already be well under way. Calls for dynamic guidelines have also been made, and platforms for such guidelines are available (5, 6). For example, the 2017 Canadian Guidelines on Opioids for Chronic Non-Cancer Pain were made available on the digital MAGICapp platform, allowing for dynamic updates in real time as the field evolves (7).

### Objectives

A methodology for dynamically updating guidelines on thyroid disease in pregnancy need to be developed. In fact, we predict that dynamic guidelines will soon become the norm. In the interim, it is important to analyze the thyroid and pregnancy literature published since the 2017 Guidelines. Specifically, the present article synthesizes the publications in two of the more controversial areas in the field of thyroid and pregnancy, namely hypothyroidism and thyroid autoimmunity. The results from the literature published in calendar years 2017 and 2018 will be placed in the context of the 2017 ATA Guideline Recommendations.

### Research Question

How does the increased rate of publications in the field of thyroid disease in pregnancy impact the state of current clinical guidelines?

## Methods

### Study Design, Search Strategy and Review Protocol

This study is conducted and reported in accordance with the Preferred Reporting Items for Systematic Reviews and Meta-Analysis (PRISMA) statement (8). One author (AD) carried out a literature search using the Pubmed database on 1/2/2019 using the terms “thyroid” and “pregnancy.” All studies with a publication date from 1/1/2017 to 12/31/2018 were identified and imported into RefWorks 2.0 (ProQuest, Ann Arbor, MI) where duplicates were removed.

**Figure 1:**
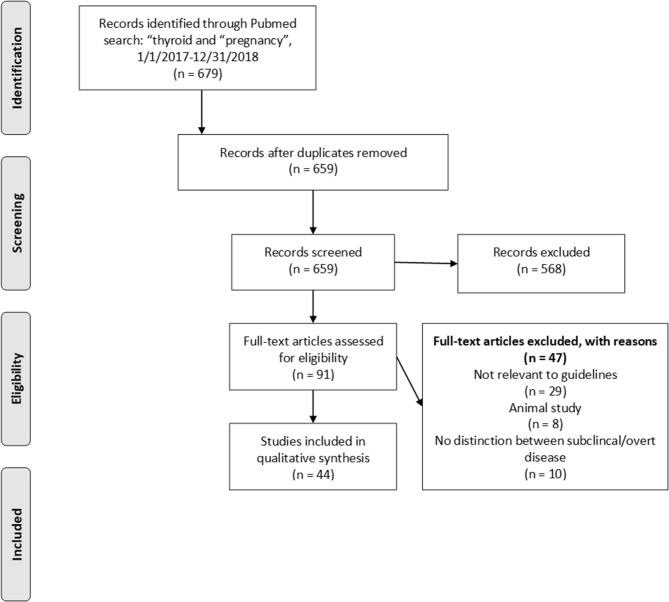
Prisma flowchart depicting article selection strategy. Articles were selected based on relevance to existing ATA recommendations or perceived need for new recommendations.

Titles and abstracts were scanned to categorize articles by subject using the subject headings of the 2017 ATA Guidelines (3). Case reports, commentaries, corrections and both narrative and systematic reviews were excluded. Articles in the categories of “Hypothyroidism and Pregnancy” and “Thyroid Auto-Antibodies and Pregnancy Complications” were reviewed in full-text by two authors (AD and AS-G). Study design and results were extracted from all articles by one author (AD). Using the published questions and recommendations in the 2017 ATA Guidelines (3), relevant articles were grouped and synthesized in this review. A flow diagram for study selection is shown in Figure 1. A list of full-text excluded articles with reasons for exclusion can be found in Supplemental Table 1.

Given the heterogeneous nature and designs of the studies included in this review, a formal, tool-based risk of bias assessment was not carried out. However, all included studies are discussed in detail within the text of this review along with any perceived limitations to their designs.

## Results

### Hypothyroidism

Sixty-six original studies were published on hypothyroidism in pregnancy since the publication of the 2017 ATA Guidelines. For this review, 26 original research studies on the effects of overt hypothyroidism, subclinical hypothyroidism and isolated hypothyroxinemia on pregnancy outcomes and treatment in pregnancy met inclusion criteria based on relevance to existing questions or recommendations posed in the 2017 ATA Guidelines.

- **Adverse Outcomes of Overt Hypothyroidism (Question 33) “What adverse outcomes are associated with overt hypothyroidism during pregnancy?”**
  Four studies were published since the 2017 ATA Guidelines that included women who were clearly overtly hypothyroid (9–12). Three out of the four studies found at least one adverse outcome associated with overt hypothyroidism (9–11). In a retrospective cohort study of 1,153 mother-child pairs from the Danish National Birth Cohort, Andersen et al. reported an association between overt hypothyroidism and lower child verbal IQ, with children born to mothers with TSH ≥10 having an average 8.9 point reduction, CI −15 to −2.4, in verbal IQ score compared to children born to mothers with a TSH of 0.1–2.49 mIU/L (9). Andersen et al. also published a case-cohort study of 7,624 pregnant women from the Danish National Birth Cohort, finding an association between maternal overt hypothyroidism and epilepsy in their children, aHR 3.5, CI 1.2–10 (10). In a prospective study of 1,082 Chinese women, Yang et al. found a significant association between overt hypothyroidism and preterm delivery with 15.4% (2/13) of mothers with overt hypothyroidism having a preterm delivery compared to 3.5% (31/882) of euthyroid mothers, aOR 8.99, CI 1.73–46.77 (11).
  On the other hand, Nelson et al. in a prospective study of 4,615 mother-child pairs, found no association between overt hypothyroidism and child educational attainment, with children from overtly hypothyroid mothers displaying no statistical difference in the number of A^*^/A grades received compared to those from euthyroid mothers, OR 0.25, CI 0.05–1.17, number of courses passed at any grade level, RR 0.95, CI 0.80–1.12, or number of courses attempted, Ratio of Geometric Means (RGM) 1.04, CI 0.92–1.18 (12).
  Two other studies included women that were labeled as “hypothyroid” without specification to overt vs. subclinical disease; however, these women were “likely” overtly hypothyroid based on the context of the studies (13, 14). Both of these studies found a link between hypothyroidism and adverse outcomes. In a population-based cohort study of 595,669 Danish children from the Danish national registry, Liu et al. (13) found a significant association between diagnosed maternal hypothyroidism during pregnancy that required eventual treatment and asthma development in children, IRR 1.16, CI 1.03–1.30. Children from mothers that did not receive treatment had an even higher risk of developing asthma, IRR 1.37, CI 1.04–1.80 (13). Jølving et al. (14) reported in a retrospective cohort study of 1,560,955 Danish children of whom 2,618 were born to mothers with Hashimoto's thyroiditis. The authors reported that the children born to women with Hashimoto's thyroiditis were more likely to develop thyroid disease themselves compared to children born to mothers without Hashimoto's thyroiditis, 2.2% (58/2,618) vs. 0.3% (4,404/1,557,577), aHR 12.83, CI 9.74–16.9. There was also an increased risk of type 1 diabetes in children born to mothers with Hashimoto's thyroiditis, 0.6% (15/2,618) vs. 0.3% (5,106/1,557,577), aHR 2.47, CI 1.46–4.18 (14).
  The 2017 ATA Guidelines conclude that there is a “clear association between overt maternal hypothyroidism and risk to the maternal-fetal unit,” specifically resulting in an increased risk of preterm delivery, low birth weight, miscarriage, and impairment of neurocognitive development their children (3). The studies reviewed above continue to find significant associations between maternal overt hypothyroidism and impaired offspring neurocognitive development and preterm delivery, as well as suggesting an increased risk of childhood asthma, thyroid disease and type I diabetes, confirming the association between maternal overt hypothyroidism and adverse maternal/fetal events.
- **Adverse Outcomes of Subclinical Hypothyroidism (Question 34) “What adverse outcomes are associated with subclinical hypothyroidism during pregnancy?”**
  Ten studies on adverse outcomes in subclinical hypothyroidism have been published in 2017 and 2018 as noted in Table 1 (9–12, 15–21). Two studies reported that subclinical hypothyroidism was associated with changes in fetal growth, however, the changes were different between studies (15, 16). In an observational cohort study of 3,988 Dutch women from the Amsterdam Born Children and Their Development (ABCD) cohort, Vrijkotte et al. found an increased risk of males being born large for gestational age in women with subclinical hypothyroidism compared to those born to women without subclinical hypothyroidism, OR 1.95, CI 1.22–3.11 (15). In a cross-sectional study of 3,832 women from the Proteomics in Pre-Eclampsia study, Carty et al. found that women with a TSH >5 mIU/L delivered lower birthweight babies than those who had a TSH <2.5 mIU/L, 3,196 vs. 3,405 gm, *P* = 0.007, but had no differences in rates of preeclampsia, miscarriage, preterm delivery or small for gestational age babies (16).
  All four studies published on subclinical hypothyroidism and preterm delivery reported a positive association (11, 17, 20, 21). In a retrospective study of 4,504 pregnant women, Arbib et al. found a higher risk of preterm delivery in women with subclinical hypothyroidism; 2% (16/796) of women with a TSH >2.5 mIU/L had a delivery prior to 34 weeks, as did 2.7% (11/404) of those with a TSH >4.0 mIU/L, compared to 1.2% (38/3,231) of euthyroid controls, *P* = 0.035 (20). In the prospective study by Yang et al. subclinical hypothyroidism was found to be associated with preterm delivery compared to euthyroid controls; 10.5% (4/38) vs. 3.5% (31/882), aOR 4.58, CI 1.46–14.4 (11). In a prospective study, Nassie et al. (17) compared 117 Israeli women presenting with preterm uterine contractions to 134 women without preterm uterine contractions. No significant difference was found between the prevalence of subclinical hypothyroidism in women with or without preterm contractions, 37% (43/117) vs. 46% (62/134), *P* = 0.13. However, the authors reported that subclinical hypothyroidism was significantly more prevalent in women with a history of prior preterm delivery. Of the 34 women with a history of preterm delivery, 62% (21/34) had subclinical hypothyroidism, compared to 41% (84/207) of women without a history of preterm delivery, *P* = 0.017 (17). Lastly, in an observational cohort study of 5,644 thyroid peroxidase antibody (TPOAb) negative women from the Dutch Generation R cohort, Korevaar et al. found that women with elevated TSH levels with higher hCG levels had a higher risk of preterm delivery and preterm premature rupture of membranes, but those who had lower levels of hCG did not, suggesting that risk of adverse events from subclinical hypothyroidism may be modified based on levels of hCG (21).
  One study reported no significant association between subclinical hypothyroidism and pregnancy loss in women with unexplained recurrent pregnancy loss. In this retrospective study of 317 Japanese women with unexplained recurrent pregnancy loss, Uchida et al. found that among women with untreated “borderline” subclinical hypothyroidism, defined as a TSH between 2.5 and 4.5 mIU/L, 29% (9/31) had a subsequent pregnancy loss compared to 17.9% (24/134) of euthyroid controls, *P* = 0.16 (18).
  One study reported an association between maternal subclinical hypothyroidism and neurodevelopmental conditions in their children. In this case-cohort study by Andersen et al. an increased risk of autism spectrum disorder (ASD) was noted, HR 1.70, CI 1.04–2.75, though no association was found between subclinical hypothyroidism and epilepsy, HR 1.04, CI 0.61–1.77 or attention deficit hyperactivity disorder (ADHD), HR 1.06, CI 0.76–1.48 (10).
  Two studies on neurodevelopmental conditions in children of mothers with subclinical hypothyroidism found no significant association (9, 12). In the retrospective study from the Danish National Birth Cohort by Andersen et al. no association was found between subclinical hypothyroidism, defined as a TSH from 2.5 to 9.99 mIU/L, and offspring verbal IQ (9). In the prospective study by Nelsen et al. no association was found between subclinical hypothyroidism and A/A^*^ grades, passing grades or courses attempted, OR 1.02, CI 0.58–1.17; RR 1.02, CI 0.80–1.12; and RGM 1.02, CI 0.92–1.18, respectively (12).
  One other study reported mixed results in regards to subclinical hypothyroidism and perinatal outcomes. In a retrospective study of 745 Japanese women, Furukawa et al. (19) found a significantly higher rate of gestational diabetes in women with subclinical hypothyroidism, 6% (10/167) vs. 0.3% (2/578) of controls, *P* < 0.01. However, in an analysis of composite rates of adverse outcomes including placental abruption, gestational diabetes, hypertension, stillbirths, babies large and small for gestational age or having low 5 min Apgars, there was no significant difference between the two groups, 14% (23/167) in women with subclinical hypothyroidism vs. 16% (92/578) of controls, *P* = 0.50 (19).
  The 2017 ATA Guidelines concluded that the evidence indicated “an increasing risk of pregnancy-specific complications, most notably pregnancy loss and preterm delivery, in relation to elevated maternal TSH concentrations” (3). The articles published in 2017 and 2018 support the 2017 ATA Guidelines' conclusion on an association between subclinical hypothyroidism and preterm delivery. The reported association appears more pronounced when subclinical hypothyroidism is defined as a TSH of 4.0 mIU/L or above, or a population-based reference value, compared to using 2.5 mIU/L or above. The association between subclinical hypothyroidism and adverse neurocognitive outcomes appears less clear. Of the three studies published on these outcomes since the 2017 ATA Guidelines, only Andersen et al. (9, 10) reported a weak association between subclinical hypothyroidism and ASD. Thus, taken as a whole, it would appear that subclinical hypothyroidism is associated with preterm delivery; however, the association with offspring neurocognitive outcomes remains controversial. Of note, the vast majority of published studies did not take into account thyroid autoimmune status in their findings. Thus, it remains difficult to draw conclusions on the effect modification thyroid autoimmunity may have on the impact of subclinical hypothyroidism on perinatal and neonatal outcomes.
- **Treatment of Subclinical Hypothyroidism (Question 37) “Should women with subclinical hypothyroidism be treated in pregnancy?” and (Recommendation 29) LT4 therapy is recommended for TPOAb-positive women with elevated TSH and TPOAb-negative women with a TSH** **>10.0 mIU/L; LT4 therapy may be considered for TPOAb-positive women with TSH** **>2.5 mIU/L or TPOAb-negative women with elevated TSH; LT4 therapy is not recommended in TPOAb-negative women with normal TSH**
  Five studies published since the 2017 ATA Guidelines addressed the treatment of subclinical hypothyroidism in pregnancy (22–26). Two studies reported a decrease in negative perinatal outcomes in women who were treated (22, 23). In a prospective study of 93 women with subclinical hypothyroidism, defined as TSH above 2.5 mIU/L in the first trimester or above 3.0 mIU/L in the second trimester, Zhao et al. (22) found a significant decrease in overall pregnancy complications, including gestational hypertension, preeclampsia, anemia and/or gestational diabetes in women treated at 8–10 weeks gestation compared to those treated at 13–16 weeks gestation or those who went untreated; 10% (3/31) vs. 41.9% (13/31) vs. 64.5% (20/31), respectively, *P* < 0.01. Similarly, rates of at least one pregnancy complication, including preterm labor, pregnancy loss, postpartum hemorrhage, or low birth weight, was decreased with early treatment of subclinical hypothyroidism, 3.2% (1/31) vs. 32.3% (10/31) vs. 38.7% (12/31), respectively, *P* = 0.03 (22). In a randomized controlled trial of 366 TPOAb negative Iranian women with subclinical hypothyroidism, defined as a TSH above 4.0 mIU/L, Nazarpour et al. found a significantly lower rate of preterm delivery in women who received levothyroxine compared to untreated women, RR 0.38, CI 0.15–0.98 (23).
  Two studies reported no benefit of treatment (24, 25). In a randomized controlled trial of 677 women, Casey et al. found no benefit of levothyroxine intervention at an average 16.7 weeks gestation compared to placebo in women with subclinical hypothyroidism, defined as TSH above the 97.5th percentile, for either IQ at 5 years of age, 97 vs. 95, *P* = 0.89, or for adverse pregnancy outcomes, including preterm delivery, 9% (31/339) vs. 11% (37/339), *P* = 0.44; preeclampsia, 6% (22/339) vs. 6% (22/338), *P* = 0.76; gestational diabetes, 7% (25/339) vs. 7% (22/338), *P* = 0.66; miscarriage 1% (4/339) vs. 2% (7/339), *P* = 0.36; and low birth weight, 10% (33/339) vs. 8% (27/338), *P* = 0.45 (24). In the 5-year follow up study of 4,609 women-child pairs from the 2012 Antenatal Thyroid Screening and Childhood Cognitive Function study, Hales et al. found no difference between the number of children with IQ <85 born to women treated for subclinical hypothyroidism at an average of 13.4 weeks gestation and those who were untreated, OR 1.15, CI 0.52–2.51 (25).
  Another study reported both benefit and harms associated with treatment of subclinical hypothyroidism in pregnancy. In a retrospective cohort study of 5,405 American women with subclinical hypothyroidism, Maraka et al. (26) reported that women who were treated with levothyroxine had significantly lower rates of pregnancy loss, 10.6% (89/843) vs. 13.5% (614/4562), *P* < 0.01. Interestingly, they reported higher rates of preterm delivery, 7.1% (60/843) vs. 5.2% (236/4562), *P* = 0.01, gestational diabetes, 12% (101/843) vs. 8.8% (401/4562), *P* = 0.02, and preeclampsia, 5.5% (46/843) vs. 3.9% (177/4562), *P* = 0.01, in women who were treated compared to those who were untreated (26).
  The 2017 ATA Guidelines conclude that the aggregate data available “appear to suggest a benefit of treatment, especially as it applies to reducing miscarriage in TPOAb-positive women.” Specifically, the ATA made a strong recommendation for the treatment of TPOAb positive women with a TSH above the pregnancy specific reference range, with a weaker recommendation for the treatment of TPOAb positive women with a TSH above 2.5 mIU/L, or TPOAb negative women with TSH above the pregnancy specific reference range. The 2017 ATA Guidelines also state that there is a probable synergistic effect of TPOAb, in that women with positive TPOAbs may suffer negative outcomes at a lower TSH value than those negative for TPOAbs (3). Whether or not treating patients with subclinical hypothyroidism prevents negative outcomes, however, remains controversial. Most studies published to date, including most of those included in this review, initiated treatment after the end of the first trimester, which may have been too late to derive benefit. In the large randomized, placebo-controlled study by Casey et al. (24), there was no benefit of treating subclinical hypothyroidism starting at a mean gestational age of 16.7 weeks. Similarly, the study by Hales et al. which began treatment at a mean gestational age of 13.4 weeks, reported no benefit of intervention (24, 25).
  Studies since the 2017 ATA Guidelines have shown that timing of intervention may play an important role in the effectiveness of intervention. Zhao et al. reported an overall reduction in pregnancy complication rate in women treated in the first trimester, but not in those treated in the second trimester (22). Similarly, the randomized controlled trial by Nazarpour et al. initiated treatment soon after the first prenatal visit, and found a reduction in the rate of preterm delivery in treated women (23).
  The 2017 ATA Guidelines state that the evidence to support their “weak recommendation,” defined by the American College of Physicians Grading System used by the ATA in their guidelines as “best action may differ based on circumstances or patients' values” or “other alternatives may be equally reasonable,” to consider treatment of TPOAb negative women with an elevated TSH is of “low-quality,” defined similarly as evidence from “observational studies/case studies.” The study by Nazarpour et al. (23), in our opinion, would meet the criteria used by the ATA for “moderate-quality evidence,” defined as “randomized controlled trial (RCT) with important limitations or strong evidence from observational studies.” Therefore, we suggest that the level of evidence for this portion of Recommendation 29 be changed from “low” to “moderate.” However, given several “high-quality,” defined as “RCT without important limitations or overwhelming evidence from observational studies,” negative studies showing no benefit of levothyroxine treatment initiated in the second trimester or later, we also suggest the recommendation for treatment of subclinical hypothyroidism in TPOAb negative women in Recommendation 29 be modified to apply only to subclinical hypothyroidism identified in the first trimester, with the addition of a “weak recommendation,” as defined in the 2017 ATA Guidelines, based on “high-quality evidence” to not treat if subclinical hypothyroidism in TPOAb negative women was diagnosed after the first trimester. Since the studies by Hales et al. (25) and Casey et al. (24) did not stratify for thyroid antibody status, further studies are still warranted before recommendations can be made for TPOAb positive women.
- **Adverse Outcomes of Isolated Hypothyroxinemia (Question 35) “What adverse outcomes are associated with isolated hypothyroxinemia in pregnancy?”**
  Ten studies on adverse outcomes in isolated hypothyroxinemia have been published since the 2017 ATA Guidelines, as noted in Table 2 (10–12, 27–33). Eight of the ten studies found an association between isolated hypothyroxinemia and adverse outcomes (10, 11, 28–33). In a retrospective study of 783 Belgian women, Furnica et al. found an association between isolated hypothyroxinemia and higher maternal BMI compared to euthyroid controls, 83.6 vs. 75.2 kg, *P* < 0.001; higher rates of breech presentation at term, 14.3% (7/49) vs. 3.6% (6/165), *P* = 0.006; higher rates of caesarian delivery, 32.7% (16/49) vs. 17.6% (29/165), *P* = 0.026; and higher rates of macrosomia 14.3% (7/49) vs. 4.9% (8/165), *P* = 0.026 (28). In an observational study of 6,031 pregnant, Chinese women in the first or third trimester of pregnancy, Zhang et al. (29) noted an association between isolated hypothyroxinemia and preeclampsia compared to euthyroid controls, with 1.35% (63/4,667) of controls developing preeclampsia compared to 3.51% (8/228) of women with hypothyroxinemia in both the first and third trimesters, aOR 2.62, CI 1.23–5.62 and 2.87% (15/523) of women with hypothyroxinemia in only the third trimester, aOR 2.16, CI 1.22–3.82. Women in the second trimester of pregnancy were not included in this study (29). In the prospective study by Yang et al. the authors reported an association between isolated hypothyroxinemia and rates of macrosomia compared to euthyroid controls, 19.6% (9/46) vs. 10.8% (95/882), aOR 2.22, CI 1.13–4.85 (11).
  Three studies linked isolated hypothyroxinemia to adverse neurocognitive effects in the offspring (10, 33). In the case-cohort study by Andersen et al. the authors reported an association between isolated hypothyroxinemia and ASD and ADHD in girls, but not boys, with 7.6% of girls born to mothers with isolated hypothyroxinemia being diagnosed with ASD compared to 1.5% of controls, and 15.2% of girls born to mothers with isolated hypothyroxinemia being diagnosed with ADHD compared to 5.8% of controls, ASD aHR 4.92, CI 2.03–11.9; ADHD aHR 2.28, CI 1.21–4.29 for girls (10). In an individual participant data meta-analysis from the Generation R, Infancia y Medio Ambiente (INMA), and Avon Longitudinal Study of Parents and Children (ALSPAC) cohorts, Levie et al. reported an association between maternal isolated hypothyroxinemia and lower verbal and non-verbal IQ of their children, OR −2.1, CI −4.8 to −0.1 and OR −3.0, CI −4.4 to −1.6, respectively, but not autistic traits (33). In a longitudinal study of 2000 women/child pairs nested within the ABCD cohort, Oostenbroek et al. reported that maternal hypothyroxinemia of <5th percentile were associated with an increased rate of teacher-reported ADHD symptoms, OR 1.70, CI 1.01–2.86, but not when criteria was extended to <10th percentile, OR 1.47, CI 0.99–2.20 (30).
  Two studies reported an association between isolated hypothyroxinemia and iron deficiency anemia; however, they found no associations with negative obstetrical or neonatal outcomes (31, 32). In a prospective study of 660 pregnant, Brazilian, TPOAb negative women, Rosario et al. found that women with iron deficiency had higher rates of isolated hypothyroxinemia of either free thyroxine (fT4) <0.92 ng/dL, <0.86 ng/dL or TT4 <7.8 ng/dL, at 20.7, 14.8, and 17.2%, respectively, compared to women without iron deficiency at 8.4, 3.9, and 6.5%, respectively, *P* = 0.038, 0.032, and 0.045, respectively, but found no associations between isolated hypothyroxinemia with other obstetrical or neonatal outcomes (32). In a prospective study of 292 pregnant, Georgian women with isolated hypothyroxinemia compared to 58 controls, Morchiladze et al. (27) reported that women with isolated hypothyroxinemia had a 41.8% (122/292) chance of having iron deficiency anemia compared to 24.1% (14/58) in the control group, RR 2.25, CI 1.13–14.53. However, they found no relationship between isolated hypothyroxinemia and miscarriage, 15.4% (45/292) vs. 10.3% (6/58), RR 1.57, CI 0.6–4.35; preterm delivery, 9.6% (28/292) vs. 6.9% (4/58), RR 1.43, CI 0.45–5.02; or premature rupture of membranes, 10.6% (31/292) vs. 5.2% (3/58), RR 2.17, CI 0.6–9.27 (31).
  Two studies reported no association between isolated hypothyroxinemia and adverse pregnancy outcomes (12, 27). In a prospective study of 104 pregnant women with isolated hypothyroxinemia compared to 58 pregnant controls, Morchiladze et al. reported no significant difference in rates of adverse pregnancy outcomes, including miscarriage, OR 1.73, CI 0.75–4.08, and preterm delivery, OR 1.28, CI 0.34–5.21 (27). In the prospective study by Nelson et al. no association was found between isolated hypothyroxinemia and A/A^*^ grades, passing grades or courses attempted, OR 0.81, CI 0.48–1.37; RR 0.96, CI 0.87–1.06; and RGM 1.00, CI 0.93–1.08, respectively (12).
  The 2017 ATA Guidelines conclude that “available evidence appears to show an association between hypothyroxinemia and cognitive development of the offspring, with uncertain effects on prematurity and low birth weight” (3). Overall, the data published since the 2017 ATA Guidelines supports this conclusion, with the large individual participant data meta-analysis by Levie et al. reporting a significant association between adverse offspring neurocognitive development and maternal isolated hypothyroxinemia (33). However, the study by Nelson et al. found no significant association between maternal isolated hypothyroxinemia and adverse educational development and achievement (12). With respect to autism spectrum disorder or attention deficit hyperactivity disorder, Levie et al. found no significant association (33). However, data from Andersen et al. suggested the gender of the child may play a role (10). Data from Oostenbroek et al. also appears to support the association between isolated hypothyroxinemia and attention deficit hyperactivity disorder (30). Little was published on the relationship between isolated hypothyroxinemia and adverse pregnancy outcomes when the 2017 ATA Guidelines were published, and this remains true today. Two studies have reported an association between iron deficiency anemia and isolated hypothyroxinemia (31, 32), however, there were a large number of tested variables, without correction for the multiple comparisons. Both studies are thus vulnerable to a high risk of false positive findings; therefore, further studies are needed. Other studies showed association between maternal isolated hypothyroxinemia with breech presentation or Cesarean section (28), preeclampsia (29) and macrosomia (11, 28), the results from these isolated observational studies require additional confirmation. Thus, current evidence continues to support the statements made in the 2017 ATA Guidelines regarding isolated hypothyroxinemia in pregnancy.
- **Treatment of Isolated Hypothyroxinemia (Question 38) “Should women with isolated hypothyroxinemia be treated with LT4 in pregnancy?” and (Recommendation 30) “Isolated hypothyroxinemia should not be routinely treated in pregnancy.”**
  One study published since the 2017 ATA Guidelines examined the treatment of isolated hypothyroxinemia in pregnancy. In the randomized controlled trial by Casey et al. there was no improvement to child IQ at 5 years of age with levothyroxine treatment of maternal isolated hypothyroxinemia compared to placebo, IQ 94 vs. 92, *P* = 0.48, nor reduction of adverse pregnancy outcomes, including preterm delivery, 12% (31/339) vs. 8% (20/339), *P* = 0.11; preeclampsia, 3% (9/339) vs. 4% (11/338), *P* = 0.64; gestational diabetes, 8% (21/339) vs. 9% (24/338), *P* = 0.62; miscarriage 1% (2/339) vs. 2% (5/339), *P* = 0.28; and low birth weight, 9% (23/339) vs. 8% (20/338), *P* = 0.68 (24).
  The 2017 ATA Guidelines conclude that there is not sufficient evidence to recommend treatment of isolated hypothyroxinemia in pregnancy. The current evidence continues to support the 2017 ATA Guideline recommendation to not treat isolated hypothyroxinemia in pregnancy.
- **Defining Criteria for Hypothyroidism (Recommendation 26) “The pregnancy-specific TSH reference range should be defined as follows: If internal or transferable pregnancy-specific TSH reference ranges are not available, an upper reference limit of** **~4.0 mIU/L may be used.”**
  Three studies published since the 2017 ATA Guidelines provide insight into the criteria by which hypothyroidism should be defined in pregnancy (16, 23, 26). In the cross sectional study by Carty et al. (16), a reduction in child birthweight was only detected in women with a TSH greater than 5.0 mIU/L; the average birthweights were in the 37th corrected percentile in women with subclinical hypothyroidism vs. the 44th corrected percentile in controls, *P* = 0.02. However, no difference was noted in those with a TSH between 2.5 and 5.0 mIU/L with the average birthweight being in the 43rd percentile, *P* = 0.536 compared to controls (16). In the randomized controlled trial by Nazarpour et al. treatment of pregnant women with subclinical hypothyroidism with levothyroxine yielded a reduction in preterm delivery only in those with a TSH >4.0 mIU/L, RR 0.38, CI 0.15–0.98, and not in those with a TSH between 2.5 and 4.0 mIU/L, RR 0.73, CI 0.33–1.76 (23). In the study by Maraka et al. (26), subgroup analysis stratified by pre-treatment TSH level suggested that the significant reduction in pregnancy loss rates was only applicable to the subgroup of women with pre-treatment TSH between 4.1 and 10 mIU/L and not those with pre-treatment TSH between 2.5 and 4.0 mIU/L, OR 0.45 [0.30–0.65] vs. OR 0.91 [0.65–1.23]. In addition, a higher rate of gestational hypertension was found in treated women with TSH between 2.5 and 4.0 mIU/L but not in those with a TSH between 4.1 and 10.0 mIU/L, OR 1.76 [1.13–2.74] vs. OR 1.48 [0.51–1.45] suggesting that treatment of subclinical hypothyroidism only reduces pregnancy loss rates in women with TSH > 4.0 mIU/L, and may result in increased harm in women treated with TSH between 2.5 and 4.0 mIU/L (26).
  The 2017 ATA Guidelines conclude that “when available, population and trimester-specific reference ranges for serum TSH during pregnancy should be defined by a provider's institute or laboratory and should represent the typical population for whom care is provided… when this goal is not feasible, pregnancy-specific TSH reference ranges obtained from similar patient populations… should be substituted… If internal or transferable pregnancy-specific TSH reference ranges are not available, an upper reference limit of ~4.0 mIU/L may be used… this represents a reduction in the nonpregnant TSH upper reference limit of ~0.5 mIU/L” (3). Studies published since the 2017 ATA Guidelines provide further confirmation of the validity of this recommendation.

**Table 1:** Studies investigating subclinical hypothyroidism and adverse maternal and child outcomes.

	**Population selection**	**TFTs**	**Definition of SCH (TSH value in mIU/L)**	**Outcome**	**Adjusted analysis**	**Finding**	**Comments**
Vrijkotte et al. (15) *N* = 3,988	Nonselected, population-based	Median 13 weeks	TSH > 2.5 mIU/L	Fetal growth	Yes	Large for gestational age in males: OR 1.95 [1.22–3.11]	Primarily a study of fT4; fT4 inversely associated with birth weight, stronger effect in males. Adjusted for TPOAb
Carty et al. (16) *N* = 3,832	Selected healthy population, population-based	12–14 weeks	2 endpoints: TSH between 2.5 and 5.0 mIU/L, TSH > 5.0 mIU/L	Pregnancy and perinatal outcomes	No	TSH > 5.0 mIU/L had lower birthweights than TSH 2.5–5 mIU/L; no other associations	Not adjusted for TPOAb
Nassie et al. (17) *N* = 251	Selected population of women presenting at a university medical center	23–24 weeks	TSH > 3.0 mIU/L	Preterm contractions	No	Higher likelihood of presence of SCH in women with history of PTD, but no association with preterm contractions	Not adjusted for TPOAb
Uchida et al. (18) *N* = 317	Selected population of women at a university hospital	Unknown timing	TSH between 2.5 and 4.5 mIU/L	Pregnancy loss	No	No association	Assessment of “borderline subclinical hypothyroidism,” does not include TSH above 4.5 mIU/L. Not adjusted for TPOAb
Furukawa et al. (19) *N* = 745	Selected population of women presenting to two private hospitals	8–20 weeks	TSH between 3.0 and 10.0 mIU/L	Pregnancy and perinatal outcomes	No	Higher rates of GDM in women with SCH, but no association with composite adverse outcomes	Adjusted for TPOAb
Arbib et al. (20) *N* = 4,504	Nonselected, population-based	<14 weeks	2 endpoints: TSH between 2.5 and 4.0 mIU/L, TSH > 4.0 mIU/L	Pregnancy and perinatal outcomes	No	Preterm delivery OR 1.81 [1.0–3.28] for TSH 2.5–4 mIU/L; OR 2.33 [1.11–1.42] for TSH > 4 mIU/L	Not adjusted for TPOAb
Andersen et al. (9) *N* = 1,153	Nonselected, population-based	5–15 weeks	TSH >97.5th % (3.09–3.85 mIU/L; gestational age specific)	Child neurodevelopment outcomes	Yes	No association	Not adjusted for TPOAb
Nelson et al. (12) *N* = 4,615	Nonselected, population-based	Median 10 weeks	TSH > 97.5th % (TSH > 2.55 mIU/L)	Child educational attainment	Yes	No association	Adjusted for TPOAb
Andersen et al. (10) *N* = 7,624	Nonselected, population-based	Median 9 weeks (Range 5–19 weeks)	TSH >97.5th % (3.09–3.85 mIU/L; gestational age specific)	Child neurodevelopmental disorders	Yes	ASD: HR 1.70, CI 1.04–2.75	No association with other neurodevelopmental disorders Not adjusted for TPOAb
Yang et al. (11) *N* = 1,082	Nonselected, population-based	Mean 27 weeks	TSH > 95th % (TSH level not reported)	Pregnancy and perinatal outcomes	Yes	Preterm delivery OR 4.58 [1.46–14.40]	Stratification by thyroid status made for small study groups and limited statistical power to detect differences between groups Not adjusted for TPOAb

*TFTs, Thyroid function tests; SCH, Subclinical hypothyroidism; TSH, Thyroid stimulating hormone; fT4, Free thyroxine; PTD, Preterm delivery; GDM, Gestational diabetes mellitus*.

**Table 2:** Studies investigating isolated hypothyroxinemia and adverse maternal and offspring outcomes.

	**Population selection**	**TFTs**	**Definition of hypothyoxinemia**	**Outcome**	**Adjusted analysis**	**Main result**	**Comments**
Morchiladze et al. (27) *N* = 350	Selected patients from a private clinic	1st Trimester	TSH 0.1–2.5 mIU/L with fT4 <10.3 pmol/L	Pregnancy and perinatal complications	No	Isolated hypothyroxinemia was associated with iron deficiency anemia, but not other pregnancy or perinatal complications	The statistical significance of only a single measured outcome (out of 15 total) raises concern for a type I error in this analysis without Bonferroni correction
Furnica et al. (28) *N* = 783	Selected patients at a university hospital	Mean 11.8 weeks	TSH 0.2–2.5 mIU/L with fT4 <5th percentile (13.0 pmol/L)	Maternal metabolic profile and pregnancy complications	No	Isolated hypothyroxinemia was associated with higher maternal BMI, as well as higher rates of breech presentation, c-section and macrosomia	
Zhang et al. (29) *N* = 6,031	Selected patients at a university affiliated hospital	9–12 weeks and 32–36 weeks	fT4 <10th percentile (13.5 pmol/L)	Pregnancy and perinatal complications	Yes	Isolated hypothyroxinemia in both the first and second trimesters was associated with preeclampsia OR 2.62 [1.23–5.62] as well as in the third trimester alone OR 2.16 [1.22–3.82]	An association between isolated hypothyroxinemia and GDM in the first trimester was not significant after adjusting for confounders
Oostenbroek et al. (30) *N* = 2,000	Nonselected, population-based	Median 13.2 weeks	fT4 <5th percentile (7.75 pmol/L)	Child problem behavior	Yes	Isolated hypothyroxinemia was associated with higher rates of teacher-reported hyperactivity/inattention, OR 1.70 [1.01–2.86], but not with higher rates of other types of behavioral problems.	Small effect size, multiple outcomes raises concern for type I error in this analysis without Bonferroni correction
Nelson et al. (12) *N* = 4,615	Nonselected, population-based	Median 10 weeks	TSH > 97.5th % (TSH > 2.55 mIU/L)	Child educational attainment	Yes	No association	
Andersen et al. (10) *N* = 7,624	Nonselected, population-based	Median 9 weeks (range 5–19 weeks)	TSH >97.5th % (3.09–3.85 mIU/L; gestational age specific)	Child neurodevelopmental disorders	Yes	Isolated hypothyroxinemia was associated with autism spectrum disorder and attention deficit hyperactivity disorder in girls but not boys.	
Morchiladze et al. (31) *N* = 162	Selected patients from a private clinic	1st Trimester	TSH 0.1–2.5 mIU/L with fT4 <10.3 pmol/L	Pregnancy and perinatal complications	No	No association	
Yang et al. (11) *N* = 1,082	Nonselected, population-based	Mean 27 weeks	TSH > 95th % (TSH level not reported)	Pregnancy and perinatal outcomes	Yes	Isolated hypothyroxinemia was associated with macrosomia OR 2.22 [1.13–4.85]	Stratification by thyroid status made for small study groups and limited statistical power to detect differences between groups
Rosario et al. (32) *N* = 660	Selected, population-based	<12 weeks	fT4 <10th percentile (0.92 ng/dL) or fT4 <5th percentile (0.86 ng/dL) or TT4 <7.8 ng/dL	Pregnancy, perinatal and neonatal outcomes	No	Isolated hypothyroxinemia was associated with higher rates of iron deficiency	The statistical significance of only a single measured outcome (out of 15 total) raises concern for a type I error in this analysis without Bonferroni correction
Levie et al. (33) *N* = 9,036	Nonselected, population-based (3 Cohorts)	<18 weeks	TSH 2.5–97.5th percentile and fT4 <2.5th percentile (cohort specific)	Child nonverbal IQ at 5–8 years of age, verbal IQ at 1.5–8 years of age, and autistic traits within the clinical range at 5–8 years of age	Yes	Isolated hypothyroxinemia was associated with a 3.9 point [−5.7, −2.2] drop in non-verbal IQ and a 2.1 point [−4.0, −0.1] drop in verbal IQ.	

*TFTs, Thyroid function tests; TSH, Thyroid stimulating hormone; fT4, Free thyroxine; BMI, Body mass index; GDM, Gestational diabetes mellitus; TT4, Total thyroxine*.

### Thyroid Autoimmunity

Twenty-six original studies were published on thyroid autoimmunity and pregnancy in 2017 and 2018. For this review, 18 studies on the relationship of thyroid autoimmunity to miscarriage; recurrent miscarriage; preterm delivery; the role of selenium supplementation in patients with autoimmunity; and thyroid autoimmunity in relationship to assisted reproductive technologies (ART), met inclusion criteria based on relevance to existing questions or recommendations posed in the 2017 ATA Guidelines. The studies examining the relationship between thyroid autoimmunity and adverse outcomes are detailed in Table 3.

- **Autoimmunity and Miscarriage (Question 16) “Is there an association between thyroid autoantibodies and spontaneous pregnancy loss in euthyroid women?”**
  Three studies found an association between thyroid autoimmunity and miscarriage (34–36). In a case-control study of 150 pregnant, euthyroid women with TPOAbs and 150 pregnant, euthyroid women without TPOAbs, Rajput et al. reported a significant increase in miscarriage in women with TPOAbs compared to those without, 12% (18/150) vs. 3.3% (5/150), respectively, *P* = 0.004 (34). Seungdamrong et al. (35) performed a secondary analysis of 1,429 women in two RCTs, the Pregnancy in Polycystic Ovarian Syndrome II study (PPCOS II) and the Assessment of Multiple Intrauterine Gestations from Ovarian Stimulation study (AMIGOS). Of the 515 women who conceived in these studies, there was a significant increase in the risk of first trimester pregnancy loss in women with a history of infertility and thyroid antibodies as compared to women with a history of infertility but without thyroid antibodies, 44% (18/41) vs. 25% (120/474), respectively, *P* = 0.02 (35). In a retrospective study of 435 Spanish women, Lopez-Tinoco et al. reported a 10-fold increase in miscarriage in women with subclinical hypothyroidism and thyroid antibodies compared to women with subclinical hypothyroidism without thyroid antibodies, 5.4% (4/74) vs. 0.6% (2/361), respectively, *P* = 0.009 (36).
  The 2017 ATA Guidelines conclude that, “Although a clear association has been demonstrated between thyroid antibodies and spontaneous pregnancy loss, this does not prove causality and the underlying mechanism for such an association remains unclear.” The articles cited above provide further confirmation of the association between early pregnancy loss and thyroid antibodies but provide no new insights into potential mechanisms.
- **Autoimmunity and Recurrent Miscarriage (Question 17) “Is there an association between thyroid antibodies and recurrent spontaneous pregnancy loss in euthyroid women?”**
  One study was published on thyroid autoimmunity in recurrent pregnancy loss. In an observational study of 74 American women with recurrent early pregnancy loss, Cueva et al. found no difference in the frequency of euploid miscarriage in women with or without thyroid autoimmunity, 42% (5/12) vs. 56% (28/50), respectively, *P* > 0.05 (37).
  The 2017 ATA guidelines conclude that, “the data for an association between thyroid antibodies and recurrent pregnancy loss are less robust than for sporadic loss.” The study by Cueva et al. (37) supports this statement. However, the presence or absence of an association remains an open question, and additional studies will be required to fully answer this question.
- **Autoimmunity and Preterm Delivery (Question 19) “Is there an association between thyroid autoantibody positivity and premature delivery?”**
  Four studies examined the association between thyroid autoimmunity and preterm delivery (34, 38–40), three of which were positive (34, 38, 40). In the study by Rajput et al. TPOAb positive women had a significant increase in preterm delivery compared to TPOAb negative women, 14% (21/150) vs. 3.3% (5/150), respectively, *P* = 0.001 (34). In a large prospective cohort study of 2,931 Chinese women, Han et al. reported a significant association between thyroid antibody positivity in the second trimester, but not the first trimester and preterm delivery, OR 1.863, CI 1.009–3.411, and, OR 1.513, CI 0.869–2.633, respectively (38). In an individual participant data meta-analysis of 11,212 women from three cohorts, the Generation R, ABCD and the Holistic Approach to Pregnancy (HAPPY) cohorts, Korevaar et al. (40) reported a significant dose-dependent relationship between TPOAb titers modified by TSH concentrations and premature delivery, *P* = 0.05 for TSH X log(TPO) interaction. This was true even for women with antibody levels considered negative by manufacturer cutoffs, especially if they also had higher levels of TSH (40). Further studies will be needed to investigate this finding.
  One study found no association between thyroid autoimmunity and preterm delivery. In a secondary analysis of the EAGeR (Effects of Aspirin in Gestation and Reproduction) randomized, placebo-controlled trial in which 1,193 women with 1–2 prior pregnancy losses were prescribed low dose aspirin or placebo, Plowden et al. found no association between thyroid antibodies and preterm delivery, aRR 1.26, CI 0.65–2.45 (39).
  The 2017 ATA guidelines conclude that “thyroid auto-antibody positivity is associated with increased risk for preterm delivery” (3). Based on the results of the four studies published since the 2017 ATA Guidelines, this statement is still appropriate, but further investigation is warranted.
- **Treatment of euthyroid autoimmunity (Question 18 and Recommendation 14) “Does treatment with LT4 or intravenous immunoglobulin therapy decrease the risk for pregnancy loss in euthyroid women with thyroid autoimmunity? and (Question 20 and Recommendation 15) “Does LT4 treatment of euthyroid women who are thyroid autoantibody positive reduce risk for premature delivery?”**
  One study since the 2017 ATA guidelines examined the impact of levothyroxine on miscarriage and preterm delivery in women with thyroid autoimmunity (45). In an Iranian randomized controlled trial comparing 56 LT4 treated, TPOAb positive women to 58 untreated, TPOAb positive women, Nazarpour et al. (45) reported a significantly lower rate of preterm delivery in treated TPOAb positive women compared to those who went untreated, 7.1% (4/56) vs. 23.7% (14/58), *P* < 0.001. There was no significant reduction in rates of miscarriage however, 3.6% (2/56) vs. 3.4% (2/54), *P* > 0.05 (45).
  This study by Nazarpour, however, included both euthyroid and subclinically hypothyroid women. In a subgroup analysis stratifying by TSH > 4 mIU/L, only treated women with TSH > 4 mIU/L showed benefit of treatment, with 5.3% (2/38) of treated women delivering preterm compared to 29.4% (10/34) of untreated women, *P* = 0.01. Women with TSH <4 mIU/L did not show benefit of treatment, 11.1% (2/18) vs. 16.7% (4/24) (45).
  The 2017 ATA guidelines concluded that “insufficient evidence exists to conclusively determine whether LT4 therapy decreases pregnancy loss risk in TPOAb-positive euthyroid women…” and “insufficient evidence exists to recommend for or against treating euthyroid pregnant women who are thyroid antibody positive with LT4 to prevent preterm delivery (3).” The study by Nazarpour et al. (45) suggests that treatment of thyroid antibody positive women with levothyroxine may decrease the risk of preterm birth; however, it is also possible that this may be limited to those women who are subclinically hypothyroid. However, the subgroup analysis of euthyroid women was very underpowered, as was the analysis on risk of miscarriage. Thus, there remains insufficient evidence to make a recommendation for or against the treatment of euthyroid women with thyroid autoimmunity in pregnancy.
- **Autoimmunity and Other Outcomes (Question 21) “Is thyroid autoimmunity in euthyroid pregnancy women associated with adverse obstetric or child outcomes other than pregnancy loss and premature birth?”**
  Five additional studies questioning an association between thyroid antibodies and preeclampsia, gestational diabetes, child metabolic abnormalities, or child IQ later in life were published since the 2017 ATA Guidelines (39, 41–44). Three studies reported a positive association (41, 43, 44). Xu et al. reported higher mean levels of thyroid antibodies in a cohort of 50 women with gestational diabetes mellitus compared to 50 women with diabetes diagnosed prior to pregnancy, TPOAbs 69.34 U/mL and thyroglobulin antibodies (TgAbs) 70.32 U/mL vs. TPOAbs 62.85 U/mL and TgAbs 64.38 U/mL, *P* = 0.021 and 0.041, respectively (41). In a retrospective analysis of 3,229 mothers and their 4,176 children, Heikkinen et al. (43) reported that TPOAbs, but not TgAbs, were associated with a higher risk of children developing metabolic syndrome, 4.8% (9/188) vs. 2.0% (77/3,921), OR 2.57, CI 1.26–5.25. TPOAbs were also associated with children having a high waist circumference, 18.3% (35/191) vs. 11.5% (456/3,954), OR 1.69, CI 1.14–2.50. Additionally, TPOAbs were associated with an increased risk of children becoming overweight or obese, 16.1% (31/193) vs. 11% (438/3,974), OR 1.56, CI 1.04–2.34 (43). In an analysis of 6,033 mother-child pairs from the ALSPAC and Generation R cohorts, Derakhshan et al. reported that TPOAbs were associated with lower mean IQ, −2.00 ± 0.92, *P* = 0.03, in the Generation R cohort, but not in the ALSPAC cohort, 0.72 ± 0.97, *P* = 0.45, after adjusting for fT4 levels (44).
  Two studies reported no association between thyroid autoimmunity and various pregnancy outcomes (39, 42). In the study by Plowden et al. the authors found no relationship between thyroid antibodies and gestational diabetes, OR 3.06, CI 0.31–30.57, or preeclampsia, OR 1.44, CI 0.37–5.59 (39). In a cross-sectional study of 64 women with either pregestational diabetes mellitus or gestational diabetes mellitus, Konar et al. reported no difference in the rate of thyroid antibody positivity between women with gestational diabetes vs. those with pregestational diabetes, 12% (6/52) vs. 25% (3/12), respectively, *P* > 0.05 (42).
  The 2017 ATA Guidelines were inconclusive on an association between thyroid antibody positivity and placental abruption, postpartum depression, and neurocognitive development (3). The literature since the 2017 ATA Guidelines add no new findings on these associations but do report a negative association between thyroid autoimmunity and preeclampsia (39), and a positive association between thyroid antibodies and gestational diabetes in one study (41), but no association in two others (39, 42). Based on this recent literature, there does not appear to be a link between thyroid antibodies and preeclampsia and the relationship between thyroid antibodies and gestational diabetes remains inconclusive; however, further investigation is warranted for both these outcomes. The two studies on childhood risk for metabolic syndrome and on childhood IQ raise intriguing questions regarding the long-term impact of thyroid antibodies on offspring development (43, 44). At present, the findings from these two studies should be considered preliminary and require confirmation. Based on the studies published since the 2017 ATA Guidelines, no alteration of the Guidelines are indicated, but further investigation is warranted.
- **Autoimmunity and Assisted Reproductive Technologies (Question 28) “Is maternal antithyroid Ab positivity associated with poorer outcomes following ART?”**
  Five studies examined the association between thyroid autoimmunity and outcomes of ART (46–50). Two studies reported a negative association between thyroid autoimmunity and outcomes after ART (48–50). In a retrospective cohort study of 300 Turkish women with diminished ovarian reserve undergoing *in-vitro* fertilization/intracytoplasmic sperm injection (IVF/ICSI), Beydilli Nacak et al. found that TPOAbs were significantly associated with “poor cycle outcome,” RR 2.8 CI 1.2–6.3 (48). In another case-control study of 52 Greek women undergoing IVF or IVF/ICSI, 26 with thyroid antibodies and 26 matched-controls, Medenica et al. reported significantly lower pregnancy rates per cycle in women with thyroid antibodies, 31% vs. 62%, *P* = 0.026 (exact cycle numbers not available), as well as significantly lower pregnancy rates per embryo transfer, 35 vs. 67%, *P* = 0.029 (exact transfer numbers not available) (50).
  However, three studies reported no difference in ART outcomes between women with and without thyroid autoimmunity (46, 47). In a retrospective cohort study of 3,143 euthyroid patients undergoing intrauterine insemination (IUI), among the 376 women who went on to conceive, Unuane et al. reported no difference in live birth rates between TPOAb positive and TPOAb negative women, 86% (19/22) vs. 82% (201/354), respectively, OR 1.05, CI 0.76–1.47 (46). In a retrospective cohort study of 123 infertile, euthyroid women undergoing IVF or IVF with ICSI, 29 with thyroid autoimmunity and 94 without, Andrisani et al. also reported no difference in implantation rates, 15 vs. 19%, *P* > 0.05 (exact cycle numbers not available), pregnancy rates, 29 vs. 34%, *P* > 0.05 (exact cycle numbers not available) or ongoing pregnancy rates, 26 vs. 31%, *P* > 0.05 (exact cycle numbers not available), although they did report significantly fewer grade 1 embryos collected in patients with antibody positivity, 22% (26/119) vs. 45% (176/394), *P* < 0.001 (47). In a case-control study of 46 Chinese women undergoing IVF with fresh embryo transfer, 19 with TPOAbs and 27 without, Lu et al. reported no difference between women with and without TPOAbs in the implantation rate, 21% (8/38) vs. 32% (17/53), *P* = 0.25 and clinical pregnancy rate, 42% (8/19) vs. 52% (14/27), *P* = 0.51 (49).
  The 2017 ATA Guidelines came to no conclusion regarding the impact of thyroid autoimmunity on ART outcomes given the mixed data available at the time (3). The studies published since the 2017 ATA Guidelines are similarly discordant. Thus, no changes to the Guidelines are warranted based on this recently published research; however, further investigation is warranted. Studies involving ART and thyroid autoimmunity suffer from differences in populations and ART protocols making aggregation of data difficult. Of note, a 2018 meta-analysis by Poppe et al. (51) noted that miscarriage rates in thyroid autoantibody positive women undergoing ART have been declining in contemporary studies compared to in studies reported in the past. The authors hypothesize that this may be due to the increase in rates of intracytoplasmic sperm injection (ICSI), which may disproportionally benefit women with thyroid autoimmunity (51). Thus, better quality studies that isolate specific ART methodologies will be needed to fully answer this question.
- **Treatment of Thyroid Autoimmunity in ART (Question 29) “Does treatment of antithyroid Ab-positive euthyroid women improve ART outcomes?” and (Recommendation 21) “Insufficient evidence exists to determine whether LT4 therapy improves the success of pregnancy following ART in TPOAb-positive euthyroid women.”**
  There has been one study published since the 2017 ATA Guidelines which evaluated treatment of euthyroid, thyroid antibody positive women undergoing ART. In an open-label, randomized trial, Wang et al. (52) compared 300 euthyroid, TPOAb positive women undergoing IVF who received levothyroxine daily, 25 μg daily for TSH <2.5 mIU/L and 50 μg daily for TSH ≥2.5 mIU/L, to 300 euthyroid women who did not receive levothyroxine. In women who conceived, the rate of miscarriage was 10% (11/107) in the treated group compared to 11% (12/113) in the control group, *P* = 0.94. Clinical pregnancy rates were 35.7% (107/300) and 37.7% (113/300), respectively, *P* = 0.61. Live birth rates were 31.7% (95/300) and 32.3% (97/300), respectively, *P* = 0.86. Preterm delivery rate was 22.1% (21/95) and 19.6% (19/97), respectively, *P* = 0.67 (52).
  The 2017 ATA Guidelines state that “based on a single small randomized clinical trial and one retrospective cohort, LT4 treatment for thyroid Ab-positive women without thyroid dysfunction undergoing IVF does not appear to improve outcomes.” However, the 2017 ATA Guideline committee concluded that these studies were insufficient to provide a recommendation for or against treatment (3). This new study by Wang et al. (52) is much larger than the previous RCT and is in support of previous results. However, the single-center design still leaves room for future confirmation in other populations. Thus, we suggest that Recommendation 21 of the ATA guidelines be updated to a “weak recommendation” that LT4 therapy in euthyroid, TPOAb positive women undergoing ART is not recommended based on “moderate-quality evidence.”
- **Selenium Supplementation for Autoimmunity (Recommendation 12) “Selenium supplementation is not recommended for the treatment of TPOAb positive women during pregnancy.”**
  In a prospective observational study of 74 Polish women, Ambroziak et al. (53) evaluated the prevalence of selenium deficiency in 29 thyroid antibody positive and 45 thyroid antibody negative women. Although the prevalence of selenium deficiency increased throughout pregnancy in both groups, selenium levels were not any different in the presence or absence of thyroid autoimmunity, giving indirect evidence to support the theory that supplementation would not benefit those with thyroid autoimmunity (53).
  The 2017 ATA Guidelines conclude that selenium supplementation should not be recommended for the treatment of TPOAb positive women during pregnancy (3). This study provides further support for this recommendation.

**Table 3:** Thyroid autoimmunity and adverse maternal and child outcomes.

	**Population selection**	**TFTs**	**Outcome**	**Adjusted analysis**	**Association with antibodies**	**Additive effects SCH**	**Comments**
Rajput et al. (34) *N* = 300	Nonselected, population-based	Median 9 weeks Range 6–12 weeks	Pregnancy and perinatal outcomes	No	TPOAb positivity associated with miscarriage (12 vs. 3.3%) and preterm delivery (14 vs. 3.3%)	Not investigated	
Seungdamrong et al. (35) *N* = 515	Nonselected, population-based	Prior to pregnancy	Live birth rate	Yes	TPOAb positivity associated with increased risk of first trimester pregnancy loss OR 2.17 [1.12–4.22] and a decreased chance of live birth OR 0.58 [0.35–0.96]	Not investigated	Study done in infertile population
Lopez-Tinoco et al. (36) *N* = 435	Nonselected, population-based	First trimester	Pregnancy and perinatal outcomes	No		TPOAb positivity associated with 10.25-fold increased risk of miscarriage in women with SCH	All study participants had SCH
Cueva et al. (37) *N* = 74	Selected recurrent pregnancy loss patients presenting at a university hospital	Prior to pregnancy	Euploid Miscarriage	No	No association	Not investigated	Thyroid autoimmunity defined as positive TPOAb and/or TgAb
Han et al. (38) *N* = 2,931	Nonselected, population-based	1st trimester (median 10 weeks) and 2nd trimester (median 26 weeks)	Preterm delivery	Yes	TPOAb positivity in both the first and second trimester was associated with early term delivery OR 1.691 [1.302–2.197]; OR 1.644 [1.193–2.264], respectively. Preterm delivery was associated with TPOAb positivity in the second trimester only OR 1.863 [1.009–3.411]	Not investigated	
Plowden et al. (39) *N* = 1,193	Selected women with previous pregnancy loss	Prior to pregnancy	Preterm delivery, gestational diabetes and preeclampsia	Yes	No association	TPOAb positive + TSH ≥ 2.5 mIU/L: no association	Low power to detect differences in women with both SCH and TPOAb
Korevaar et al. (40) *N* = 11,212	Nonselected, population-based	<20 weeks	Preterm delivery	Yes	TPOAb positivity associated with preterm delivery in dose-dependent manner	Increasing TSH associated with dose-dependent higher risk of preterm delivery in women with TPOAb positivity	TPOAb associated with preterm delivery even at levels below the manufacturer cutoffs
Xu et al. (41) *N* = 50	Selected pregnant women with gestational diabetes and diabetes	24–28 weeks	Gestational diabetes and diabetes	No	TPOAb more prevalent in women with GDM compared to women with nongestational diabetes	Not investigated	
Konar et al. (42) *N* = 64	Selected pregnant women with gestational diabetes and diabetes	Not specified	Gestational diabetes and diabetes	No	No association	Not investigated	
Heikkinen et al. (43) *N* = 3,229	Nonselected, population-based	Mean 10.7 weeks	Cardiometabolic risk factors in children	Yes	TPOAb positivity associated with higher risk of metabolic syndrome, high waist circumference and becoming overweight/obese	Not investigated	TgAb also tested, but no associations found
Derakhshan et al. (44) *N* = 6,033	Nonselected, population-based	≤ 18 weeks	Child IQ at 5–10 years	Yes	TPOAb positivity associated with lower mean child IQ in Generation R cohort, but not ALSPAC cohort.	Not investigated	Adjustment for TSH or fT4 did not affect results

*TFTs, Thyroid Function Tests; SCH, Subclinical hypothyroidism; TPOAb, Thyroid peroxidase antibody; TgAb, Thyroglobulin antibody; GDM, Gestational diabetes mellitus; ALSPAC, Avon Longitudinal Study of Parents and Children; TSH, Thyroid stimulating hormone; fT4, Free thyroxine*.

## Discussion

### Summary of Main Findings

Guidelines published by most medical professional organizations are static documents. For example, the ATA published their initial thyroid and pregnancy guidelines in 2011 and generated an updated document in 2017. With the release of new data, guidelines may no longer reflect best evidence. This problem is directly related to the length of time between guideline release. What is needed is a paradigm shift from static guidelines published every 5–6 years, to dynamic guidelines which are modified on an annual basis, or as soon as evidence is published. With state-of-the-art online publishing tools, dynamic guidelines can be seamlessly updated and disseminated. Such platforms have been developed, but are not yet in widespread use (5). This systematic review highlights the importance of dynamic guidelines through the lens of associations between hypothyroidism and/or thyroid autoimmunity and reproduction.

Although the majority of manuscripts published since the 2017 ATA Guidelines reviewed in this paper support the ATA Guideline Recommendations, there are two areas where we believe the new literature necessitates changes. We suggest that Recommendation 29 of the 2017 ATA Guidelines be changed to recommend against treatment of subclinical hypothyroidism diagnosed after the first trimester in women who are TPOAb negative; we also recommend that the level of evidence for the section of Recommendation 29 supporting the treatment of TPOAb negative women with elevated TSH levels be modified to “moderate-quality evidence” and the statement itself be qualified to apply only to women diagnosed in the first trimester or earlier. We also suggest that Recommendation 21 of the 2017 ATA Guidelines be changed to recommend against the treatment of euthyroid, TPOAb positive women undergoing ART. The exact language of the suggested changes is presented in Table 4.

**Table 4:** Proposed changes to the ATA 2017 recommendations with supporting studies published in 2017 and 2018.

**ATA 2017 recommendation 29**	**Proposed changes (Underlined)**
LT4 therapy is recommended for – TPOAb-positive women with a TSH greater than the pregnancy-specific reference range **Strong recommendation, moderate-quality evidence** – TPOAb-negative women with a TSH greater than 10.0 mIU/L **Strong Recommendation, low quality evidence**	LT4 therapy is recommended for – TPOAb-positive women with a TSH greater than the pregnancy-specific reference range **Strong recommendation, moderate-quality evidence** – TPOAb-negative women with a TSH greater than 10.0 mIU/L **Strong Recommendation, low quality evidence**
LT4 therapy may be considered for – TPOAb-positive women with TSH concentrations >2.5 mIU/L and below the upper limit of the pregnancy-specific reference range **Weak recommendation, moderate-quality evidence** – TPOAb-negative women with TSH concentrations greater than the pregnancy-specific reference range and below 10.0 mIU/L **Weak recommendation, low-quality evidence** LT4 therapy is not recommended for – TPOAb-negative women with a normal TSH **Strong recommendation, high-quality evidence**	LT4 therapy may be considered for – TPOAb-positive women with TSH concentrations >2.5 mIU/L and below the upper limit of the pregnancy-specific reference range **Weak recommendation, moderate-quality evidence** – TPOAb-negative women with TSH concentrations greater than the pregnancy-specific reference range and below 10.0 mIU/L diagnosed in the first trimester or earlier **Weak recommendation**, moderate**-quality evidence** LT4 therapy is not recommended for – TPOAb-negative women with a normal TSH **Strong recommendation, high-quality evidence** – TPOAb-negative women with TSH concentrations > 2.5 mIU/L and below 10.0 mIU/L diagnosed in the second trimester or later Weak recommendation, high-quality evidence
**Supporting studies**	**Finding**
Casey et al. (24)	No difference in child IQ at 5 years or adverse pregnancy outcomes in treated vs. untreated women with subclinical hypothyroidism in pregnancy. Treatment initiated in 2nd trimester.
Hales et al. (25)	No difference in child IQ at 9.5 years in treated vs. untreated women with subclinical hypothyroidism in pregnancy. Treatment initiated in 2nd trimester.
Nazarpour et al. (23)	Significant reduction in preterm delivery rate in treated TPOAb-negative women with TSH > 4.0 mIU/L and normal fT4 index compared to untreated women. Treatment initiated soon after first prenatal visit.
**ATA 2017 recommendation 21 section**	**Proposed changes (Underlined)**
Insufficient evidence exists to determine whether LT4 therapy improves the success of pregnancy following ART in TPOAb-positive euthyroid women. However, administration of LT4 to TPOAb-positive euthyroid women undergoing ART may be considered given its potential benefits in comparison to its minimal risk. In such cases, 25–50 μg of LT4 is a typical starting dose. **Weak recommendation, low-quality evidence**	LT4 therapy is not recommended for TPOAb-positive euthyroid women undergoing ART. Weak recommendation, moderate-quality evidence
**Supporting studies**	**Finding**
Wang et al. (52)	No difference in miscarriage rate, clinical pregnancy rate, live birth rate or preterm delivery rate between levothyroxine treated, TPOAb-positive euthyroid women undergoing ART and those who received no treatment.

*Changes are highlighted by underlined text*.

*ATA, American Thyroid Association; LT4, Levothyroxine; TPOAb, Thyroid peroxidase antibody; TSH, Thyroid stimulating hormone; fT4, Free thyroxine; ART, Assisted reproductive technology*.

Another example of why dynamic guidelines are needed is that since the completion of this systematic review, the landmark, double-blind, Randomized Controlled Trial of the Efficacy and Mechanism of Levothyroxine Treatment on Pregnancy and Neonatal Outcomes in Women with Thyroid Antibodies (TABLET) trial of 940 euthyroid, English women with a history of infertility or miscarriage was published by Dhillon-Smith et al. (54) in March of 2019. The investigators reported no difference in live birth rates between the treatment group vs. the placebo group, 37% (176/470) vs. 38% (178/470), respectively, RR 0.97, 95% CI 0.83–1.14 (54). Recommendation changes based on this important RCT should not await the third version of the ATA Guidelines. The fact that this study was published in the midst of the writing of this review only serves to further highlight the need for more dynamically adaptable guideline creation policies.

Presently, there are 14 ongoing clinical trials throughout the world on thyroid and pregnancy, based on registration at Clinicaltrials.gov. As these clinical trials are published, dynamic guidelines would allow for updated recommendations to be made immediately.

### Limitations

The authors of this systematic review do not represent the ATA nor any other professional organization. We recognize that our conclusions on the recent literature represent our opinions alone and should be interpreted accordingly. Although we readily acknowledge this limitation, we reiterate that in addition to systematically reviewing the recent literature, we sought to illustrate the need for dynamic guidelines.

We also recognize that there are arguments to be made against dynamic guidelines, including that changing guidelines too quickly may result in difficulty for the general practicing physician to keep pace with rapid changes in practice guidelines. Nevertheless, we believe that the benefits of creating a well thought out dynamic guideline development process outweighs the potential downsides.

## Conclusions

How to best create a process for the generation and continual updating of dynamic guidelines should be a high-priority topic for organizations such as the ATA. Based on the quality of the evidence, recommendations should be reviewed and updated. A dynamic guideline committee would need to convene frequently. Perhaps membership of the committee would be rotational, with one-fifth of the committee changing within any given year. This would provide both consistency over time, along with the inclusion of new members with developing expertise. In conclusion, through the introduction of a dynamic guideline process, the ATA would be able to provide optimal information to practicing clinicians on a real time basis.

## Author Contributions

AS-G conceived of the presented idea. AD performed the literature search. AD and AS-G reviewed the literature. AS-G, AD, and MS contributed to writing and editing of the manuscript.

### Conflict of Interest

The authors declare that the research was conducted in the absence of any commercial or financial relationships that could be construed as a potential conflict of interest.
