# Supplementary material for: The Need for Dynamic Clinical Guidelines: A Systematic Review of New Research Published After Release of the 2017 ATA Guidelines on Thyroid Disease During Pregnancy and the Postpartum
Source: Front Endocrinol (Lausanne). 2020 Apr 7;11:193. doi: 10.3389/fendo.2020.00193 (PMC7154179; doi:10.3389/fendo.2020.00193)
Supplement: Supplementary file 1 [file Table_1.docx]

**Supplemental Table 1. Articles excluded after full text review.**

| **Author** | **Title** | **Journal** | **Exclusion Reason** |
| --- | --- | --- | --- |
| Wang et al. 2017 | Urinary iodine in early pregnancy is associated with subclinical hypothyroidism in Tianjin, China: an observational study. | BMC Endocr Disord | Not relevant to guidelines* |
| Johns et al. 2017 | Longitudinal Profiles of Thyroid Hormone Parameters in Pregnancy and Associations with Preterm Birth | PLOS ONE | No distinction between subclinical hypothyroidism and overt hypothyroidism |
| Blumenthal & Eastman 2017 | Beneficial Effects on Pregnancy Outcomes of Thyroid Hormone Replacement for Subclinical Hypothyroidism | Journal of Thyroid Research | No distinction between subclinical hypothyroidism and overt hypothyroidism |
| Kumar et al. 2018 | Mechanisms involved in epigenetic down-regulation of Gfap under maternal hypothyroidism | Biochem Biophys Res Commun | Not relevant to guidelines |
| Nazarpour et al. 2018 | Validation of Billewicz Scoring System for Detection of Overt Hypothyroidism During Pregnancy | Int J Endocrinol Metab | Not relevant to guidelines |
| Castillo Lara et al. 2017 | Hypothyroidism screening during first trimester of pregnancy | BMC Pregnancy Childbirth | No distinction between subclinical hypothyroidism and overt hypothyroidism |
| Akter et al. 2018 | Thyroid Dysfunction and Autoimmunity in First Trimester of Pregnancy, Single Center Experience in Bangladesh | Mymensingh Med J | Not relevant to guidelines |
| Gilbert et al. 2017 | Adult hippocampal neurogenesis is impaired by transient and moderate developmental thyroid hormone disruption | Neurotoxicology | Not relevant to guidelines |
| Melancia et al. 2017 | Testing the correlation between experimentally-induced hypothyroidism during pregnancy and autistic-like symptoms in the rat offspring | Behav Brain Res | Animal study |
| Strobl et al. 2017 | Opposing Effects of Maternal Hypo- and Hyperthyroidism on the Stability of Thalamocortical Synapses in the Visual Cortex of Adult Offspring | Cereb Cortex | Not relevant to guidelines |
| Cai et al. 2017 | Outcome of in vitro fertilization in women with subclinical hypothyroidism | Reprod Biol Endocrinol | Not relevant to guidelines |
| Caliskan et al. 2017 | Subclinical hypothyroidism: Is it important in intracytoplasmic sperm injection cycles | Turk J Obstet Gynecol | Not relevant to guidelines |
| Harris et al. 2017 | Hypothyroidism in utero stimulates pancreatic beta cell proliferation and hyperinsulinaemia in the ovine fetus during late gestation | J Physiol | Animal study |
| Triggianese et al. 2017 | Systemic Sclerosis: Exploring the Potential Interplay Between Thyroid Disorders and Pregnancy Outcome in an Italian Cohort | Isr Med Assoc J | Not relevant to guidelines |
| Endendijk et al. 2017 | Maternal thyroid hormone trajectories during pregnancy and child behavioral problems | Horm Behav | No distinction between subclinical hypothyroidism and overt hypothyroidism |
| Sullivan et al. 2017 | Randomized Trial Comparing Two Algorithms for Levothyroxine Dose Adjustment in Pregnant Women With Primary Hypothyroidism | J Clin Endocrinol Metab | Not relevant to guidelines |
| Kaduskar et al. 2017 | Prepregnancy Hypothyroidism versus Gestational Hypothyroidism: A Comparative Study | Indian J Endocrinol Metab | Not relevant to guidelines |
| Neelaveni et al. 2017 | Postpartum Follow-up in Women Diagnosed with Subclinical Hypothyroidism during Pregnancy | Indian J Endocrinol Metab | Not relevant to guidelines |
| Moog et al. 2017 | Childhood maltreatment is associated with increased risk of subclinical hypothyroidism in pregnancy. | Psychoneuroendocrinology | Not relevant to guidelines |
| Hassan et al. 2017 | Neurodevelopment and Thyroid Hormone Synthesis Inhibition in the Rat: Quantitative Understanding Within the Adverse Outcome Pathway Framework | Toxicol Sci | Animal study |
| Akram et al. 2017 | Incidence of Subclinical Hypothyroidism and Hypothyroidism in Early Pregnancy | J Womens Health (Larchmt) | Not relevant to guidelines |
| Miko et al. 2017 | Characteristics of peripheral blood NK and NKT-like cells in euthyroid and subclinical hypothyroid women with thyroid autoimmunity experiencing reproductive failure | J Reprod Immunol | Not relevant to guidelines |
| Sofronova et al. 2017 | Antenatal/early postnatal hypothyroidism alters arterial tone regulation in 2-week-old rats | J Endocrinol | Animal study |
| Alcigir et al. 2017 | Neuroprotective activity of cannabinoid receptor-2 against oxidative stress and apoptosis in rat pups having experimentally-induced congenital hypothyroidism | Dev Neurobiol | Animal study |
| Collares et al. 2017 | Maternal thyroid function, prepregnancy obesity and gestational weight gain-The Generation R Study: A prospective cohort study | Clin Endocrinol (Oxf) | No distinction between subclinical hypothyroidism and overt hypothyroidism |
| Kalra et al. 2018 | Prevalence of Hypothyroidism in Term Pregnancies in North India | Indian J Endocrinol Metab | Not relevant to guidelines |
| Komendova et al. 2018 | Intellectual performance of children of mothers with an untreated thyroid disorder in the first trimester of pregnancy | Endokrynol Pol | No distinction between subclinical hypothyroidism and overt hypothyroidism |
| Kawahori et al. 2018 | Mild Maternal Hypothyroxinemia During Pregnancy Induces Persistent DNA Hypermethylation in the Hippocampal Brain-Derived Neurotrophic Factor Gene in Mouse Offspring | Thyroid | Animal study |
| Getahun et al. 2018 | Association between maternal hypothyroidism and autism spectrum disorders in children | Pediatr Res | No distinction between subclinical hypothyroidism and overt hypothyroidism |
| Harder et al. 2018 | Maternal thyroid hormone is required for parvalbumin neurone development in the anterior hypothalamic area | J Neuroendocrinol | Not relevant to guidelines |
| Barisic et al. 2018 | Higher levels of thyrotropin in pregnancy and adverse pregnancy outcomes | J Matern Fetal Neonatal Med | No distinction between subclinical hypothyroidism and overt hypothyroidism |
| Pakkila et al. 2018 | Maternal Thyroid Function During Pregnancy and the Child's Linguistic and Sensory Development in the Northern Finland Birth Cohort 1986 | Front Endocrinol (Lausanne) | No distinction between subclinical hypothyroidism and overt hypothyroidism |
| Johns et al. 2018 | Subclinical Changes in Maternal Thyroid Function Parameters in Pregnancy and Fetal Growth | J Clin Endocrinol Metab | Not relevant to guidelines |
| Leger et al. 2018 | Developmental milestones at one year for the offspring of mothers with congenital hypothyroidism: a population-based study | Eur J Endocrinol | Not relevant to guidelines |
| Pop et al. 2018 | Longitudinal Trajectories of Gestational Thyroid Function: A New Approach to Better Understand Changes in Thyroid Function | J Clin Endocrinol Metab | Not relevant to guidelines |
| Petersen et al. 2018 | Maternal thyroid disorder in pregnancy and risk of cerebral palsy in the child: a population-based cohort study | MBMC Pediatr | No distinction between subclinical hypothyroidism and overt hypothyroidism |
| Haensgen et al. 2018 | Gestational Hypothyroxinemia Affects Its Offspring With a Reduced Suppressive Capacity Impairing the Outcome of the Experimental Autoimmune Encephalomyelitis | Front Immunol | Not relevant to guidelines |
| Gaynullina et al. 2018 | Antenatal/early postnatal hypothyroidism increases the contribution of Rho-kinase to contractile responses of mesenteric and skeletal muscle arteries in adult rats | Pediatr Res | Animal study |
| Xia et al. 2018 | Effect of maternal hypothyroidism during pregnancy on insulin resistance, lipid accumulation, and mitochondrial dysfunction in skeletal muscle of fetal rats | Biosci Rep | Animal study |
| Xiu et al. 2018 | miRNA-125b-5p Suppresses Hypothyroidism Development by Targeting Signal Transducer and Activator of Transcription 3 | Med Sci Monit | Not relevant to guidelines |
| Promberger et al. 2017 | A Retrospective Study on the Association between Thyroid Autoantibodies with β2-glycoprotein and Cardiolipin Antibodies in Recurrent Miscarriage | Iran J Allergy Asthma Immunol | Not relevant to guidelines |
| Adrisani et al. 2018 | The influence of thyroid autoimmunity on embryo quality in women undergoing assisted reproductive technology | Gynecol Endocrinol | Not relevant to guidelines |
| Mintziori et al. 2017 | Thyroid function and autoimmunity during ovarian stimulation for intracytoplasmic sperm injection | Reprod Fertil Dev | Not relevant to guidelines |
| Bliddal et al. 2017 | Increase in thyroglobulin antibody and thyroid peroxidase antibody levels, but not preterm birth-rate, in pregnant Danish women upon iodine fortification | Eur J Endocrinol | Not relevant to guidelines |
| Yehuda et al. 2017 | Parity and Risk of Thyroid Autoimmunity Based on the NHANES (2001-2002, 2007-2008, 2009-2010, and 2011-2012) | J Clin Endocrinol Metab | Not relevant to guidelines |
| Chaudhary et al. 2017 | Iodine and Thyroid Function Status, and Anti-thyroid Peroxidase Antibody among Pregnant Women in Eastern Nepal | J Nepal Health Res Counc | Not relevant to guidelines |
| Wesseloo et al. 2018 | Thyroid peroxidase antibodies during early gestation and the subsequent risk of first-onset postpartum depression: A prospective cohort study. | J Affect Disord | Not relevant to guidelines |

*Not relevant to guidelines refers to articles that do not answer questions posed by the ATA 2017 guidelines in Section V – Thyroid Autoantibodies and Pregnancy Complications or Section VII – Hypothyroidism and Pregnancy.
